# Use of Deep Eutectic Solvents in Polymer Chemistry–A Review

**DOI:** 10.3390/molecules24213978

**Published:** 2019-11-03

**Authors:** Michal Jablonský, Andrea Škulcová, Jozef Šima

**Affiliations:** 1 Institute of Natural and Synthetic Polymers, Department of Wood, Pulp and Paper, Faculty of Chemical and Food Technology, Slovak University of Technology in Bratislava, Radlinskeho 9, Bratislava SK-812 37, Slovakia; michal.jablonsky@stuba.sk; 2 Department of Zoology and Fisheries, Faculty of Agrobiology, Food and Natural Resources, Czech University of Life Science, Kamýcka 129, 165 00 Prague 6–Suchdol, Czech Republic; 3 Institute of Chemical and Environmental Engineering, Department of Environmental Engineering, Faculty of Chemical and Food Technology, Slovak University of Technology in Bratislava, Radlinskeho 9, Bratislava SK-812 37, Slovakia; 4 Department of Inorganic Chemistry, Faculty of Chemical and Food Technology, Slovak University of Technology in Bratislava, Radlinskeho 9, Bratislava SK-812 37, Slovakia; jozef.sima@stuba.sk

**Keywords:** deep eutectic solvents, polymerization, modification, polymer, cellulose

## Abstract

This review deals with two overlapping issues, namely polymer chemistry and deep eutectic solvents (DESs). With regard to polymers, specific aspects of synthetic polymers, polymerization processes producing such polymers, and natural cellulose-based nanopolymers are evaluated. As for DESs, their compliance with green chemistry requirements, their basic properties and involvement in polymer chemistry are discussed. In addition to reviewing the state-of-the-art for selected kinds of polymers, the paper reveals further possibilities in the employment of DESs in polymer chemistry. As an example, the significance of DES polarity and polymer polarity to control polymerization processes, modify polymer properties, and synthesize polymers with a specific structure and behavior, is emphasized.

## 1. Deep Eutectic Solvents in Polymer Chemistry

Starting with the pioneering works of Abbott and his group, a new kind of two- or more-component solvents has emerged, named deep eutectic solvents (DESs) [1,2]. It has been documented by several research teams that DESs can be considered as green solvents [3,4,5,6,7,8]. The components of DESs are usually organic compounds frequently produced by biotechnological processes. Along with their involvement in chemical processes, the employment of DESs in biochemical and biotechnological processes has been clearly documented in several papers and its increase can be justifiably expected [9,10,11,12,13,14]. Most DESs are biodegradable and non-toxic. This review deals with selected specific aspects related to synthetic polymers, polymerization processes producing such polymers, and natural cellulose-based nanopolymers. Particularly, the role of DESs in the mentioned aspects of polymer chemistry is evaluated.

## 2. Meaning of the Terms Used

In the two interconnected fields examined here, green solvents and polymers, rapid developments have been recorded, thus making it important to establish the precise meaning of the terms used. This chapter is aimed at explaining the meaning of the basic terms used, as understood in this paper.

Based on the environmental assessment of solvents that covers major aspects of the environmental performance of solvents in chemical production, as well as important health and safety issues, a group of environmentally friendly solvents, or bio-solvents, which are derived from the processing of agricultural crops has been termed green solvents. Green solvents, mainly bio-derived ones, were developed as a more environmentally friendly alternative to petrochemical solvents [15,16,17,18,19].

Of all the kinds of green solvents, this review focuses in particular on deep eutectic solvents (DESs). To ensure better consistency of the terms green solvents and DESs, of all four classes of DESs, this review will deal just by that not containing metal compounds, i.e., Class III in Table 1. Deep eutectic solvents are mixtures of two or more components—a hydrogen bond donor (HBD) and a hydrogen bond acceptor (HBA)—which can bond with each other to form a eutectic mixture, having a final melting point that is lower than the melting point of the HBD and HBA raw materials, thus becoming liquid at room temperature [20,21]. When the compounds that constitute the DES are exclusively primary metabolites, namely, amino acids, organic acids, sugars, or choline derivatives, the DESs are called natural deep eutectic solvents (NADESs). With regard to mixtures with low melting temperature, the terms low-transition temperature mixtures (LTTMs) and low-melting mixtures (LMMs) are also used. Comparing the definitions of DESs, LTTMs and LMMs, it is obvious that there is no substantial difference among them and therefore we will use the acronym DESs to refer to all the above-mentioned low melting mixtures [11,22,23]. It is worth noting that DESs should be distinguished from room temperature ionic liquids (ILs) made of ions (ionic pairs). In fact, DESs and ILs share many common interesting properties, such as low volatility, wide liquid range, high thermal stability and conductivity. Their main difference is that ILs are formed through ionic bonds between ionic components. DESs are prepared by mixing two solids capable of interacting via hydrogen bonds [24,25].

A polymer is a synthetic or naturally occurring substance that has a molecular structure built up chiefly or completely from a large number of similar units bonded together. Polymers containing only a single type of repeat units are known as homopolymers, while polymers containing two or more types of repeat units are called copolymers.

Cellulose is a polysaccharide consisting of a linear, somewhat rigid chain of several hundred to more than 10,000 D-glucose units linked by β-1,4 bonds. This bonding motif differs from the α-1,4 glucose linkage of starch, which has a coiled chain structure.

Nanocellulose is cellulose consisting of particles possessing at least one dimension less than 100 nanometres. In general, this term covers different types of cellulosic materials such as cellulose nanocrystals (CNC), nanofibrillated cellulose (NFC), cellulose nanofibrils (CNF), microfibrillated cellulose (MFC), carboxymethylated cellulose (CMC), microcrystalline cellulose (MCC), cellulose filaments (CF), and cellulose whiskers [26]. Nanocellulose is derived from different types of lignocellulosic sources, such as wood, cellulose fibres from cotton, hemp, wheat straw, sisal, hemp, bleached pulps, while the main source remains wood pulp. It can be prepared by two principal ways. One of them is a chemical process, acid hydrolysis, giving rise to highly crystalline and rigid nanoparticles, which are shorter (10^2^‒10^3^ nm). The other lies in mechanical procedures comprising homogenization, microfluidization or grinding [27,28,29]. Although there are many papers investigating nanocellulose, they rarely mention the real or potential use of DESs in the preparation of nanocellulose (see, e.g., [26]). This work tries to fill the gap.

## 3. Preparation, Composition and Properties of Deep Eutectic Solvents

### 3.1. Preparation of Deep Eutectic Solvents

DESs are usually prepared by the following four methods:

- The heating method is the most commonly used; it consists of mixing and heating the compounds at approximately 100 °C under constant stirring until forming a clear homogeneous liquid, which can usually take about 60 to 90 min [30].
- The grinding method is based on mixing the compounds at room temperature and grinding them until a clear liquid is formed [31].
- Evaporation method: using a rotary device, the components are dissolved in water and evaporated at 50 °C. The liquid obtained is kept in a silica gel desiccator until a constant weight is obtained [32].
- Freeze-drying method: this method consists in the lyophilisation of an aqueous solution consisting of individual DES components. However, this method is rarely used in comparison with the first three mentioned ones [33]. Water has been detected in the freeze-dried mixture, since it tends to interact with DES components and eventually becomes a part of the DES network. Being bound to DES components, water cannot fully be removed in this case.

Thus, different methods to prepare a DES can lead to different properties in the final product.

### 3.2. Classification of DESs

Eutectic solvents generally consist of two or three components that are capable of joining together through hydrogen bonding interactions to form a eutectic mixture. The resulting DES is characterized by a significantly lower melting temperature (usually below 150°C) than that of its the individual components. In most cases, a DES is obtained by mixing a quaternary ammonium salt with metal salts or a hydrogen bond donor (HBD), which has the ability to form a complex with the halide anion of quaternary ammonium salt. In general, four types of eutectic solvents are distinguished [34].

One of the most widespread components used to prepare DESs is choline chloride (ChCl). ChCl is a cheap, biodegradable and non-toxic quaternary ammonium salt that can either be extracted from biomass or easily synthesized from fossil fuels. As far as its price is concerned, high-quality 99% food grade or 99% pharmaceutical grade choline chloride is supplied for 10–30 $/kg; industrial 98% choline chloride is sell for 5–9 $/kg at minimal order 20 kg (according to data available at www.alibaba.com). This non-toxic salt is an essential nutrient [35] and is used in treatment of several diseases [36]. In combination with safe hydrogen bond donors such as urea, renewable carboxylic acids, polyols, imidazole derivatives, amides, DESs can be formed. In biochemical and biotechnological processes it is an advantage to avoid the presence of a metal compound in the system and thus, mainly DESs of type III are exploited. It is worth noting that both binary and ternary DESs have found their application in many fields [11], although the literature provides no detailed characterization nor determination of their physical parameters. The most common two-component DESs used, for which at least one parameter is described in the literature, as well as their physical parameters, are gathered in Table 2.

As for ternary DESs, their successful employment in various chemical and physical processes has been experienced, although not at least their freezing point or glass transition temperature was determined. Literature reports on the value of at least another quantity are extremely rare. The composition and characterization of a few of the ternary DESs described in the literature are listed in Table 3.

### 3.3. Physical and Chemical Properties

The physico-chemical properties of DESs are clearly affected by numerous factors, the most important of which seem to be the nature of both the HBA and HBD, their molar ratio, the temperature and the water content. Consequently, deep eutectic solvents can be tailored according to desired applications [31]. The physicochemical properties of DESs have been widely explored because of the need to accurately exploit the data in various industrial applications. The key features of DESs include their freezing point, density, viscosity, surface tension, conductivity, thermal stability, polarity, acidity [11,34,39,40,41]. Based on literary sources, we tried to characterize some systems and their physical and chemical properties. Unfortunately, in most of the published works, these systems are not characterized in terms of all these properties. In the majority of published papers, even such important parameter as the sharp freezing point for crystalline compounds (or glass transition temperature in cases of amorphous compounds) is missing, while the papers are focused on the practical applicability of DESs rather than on their detailed theoretical investigation. In addition, apart from temperature dependences of density, viscosity and conductivity [11,34], the interrelationships of the mentioned properties, such as viscosity-density dependences, are very rarely (if ever) discussed in the literature. 

One of the reasons of the absence of a detailed theoretical investigation may lie in the fact that one can find several values of characteristic properties for an investigated DES, which impedes finding a mutual correlation. Even for the DESs examined and described in the most detailed way, namely, those composed of choline chloride and urea in the 1:2 molar ratio, the value of viscosity at 303 K available in the literature ranges from 152 to 527 mPa·s, and that of density from 1.1887 to 1.26 g·cm^−3^ [42]. It may be a consequence of several factors, one of them relates to the purity of the original constituents, particularly the content (traces) of water. In any case, it is remarkable that water is not included in the classification of DESs (Table 1), although it is certainly significantly involved in the formation of hydrogen bonding, which is the basic chemical characteristic of DESs (Table 1), although it is certainly significantly involved in the formation of hydrogen bonding, which is the basic chemical characterization of DES [31]. As for the mentioned individual physical properties, no correlation was observed between the freezing point (glass transition temperature) of the eutectic mixture and the melting points of the free components. Most of the DESs are highly viscous at room temperature (>100 cP). This is most probably due to the hydrogen bond network between the components. The viscosity of a eutectic mixture is also affected by the nature of its components, the HBA/HBD molar ratio, the temperature, and the water content [31].

DESs usually exhibit higher densities than water, varying between 1.04 and 1.63 g·cm^−3^ at 298 K and are highly affected by the HBA/HBD molar ratio. The presence of water usually affects the density of DESs only slightly [31]. Generally, most DESs present low ionic conductivities (<2 mS·cm^−1^ at room temperature) due to their high viscosity. So, when increasing the temperature, the viscosity decreases and the ionic conductivity increases. The addition of water also increases the ionic conductivity of DESs [31]. Of the chemical properties, the acido-basic ones have been observed most often. The pH of a standard 1 mol·L^−1^ aqueous solution of DES has been measured to be below 7 [11].

In the systems consisting of a DES and a solid polymer, they exert mutual influence on their interface. Along with the above-mentioned physical properties of DESs, another property of both DES and polymer should be taken into account. This property is polarity. In most published review papers, polarity is not discussed. Because of this absence and the relevance of polarity in polymer chemistry, we will dwell upon this parameter in the following section.

### 3.4. Polarity of Deep Eutectic Solvents and Polymers

To express the polarity of chemical substances and their mixtures, in particular solvents including DESs, several paremeters are used. A classic, most frequently used quantity is permittivity (dielectric constant) [43] and spectral parameter ET(30). For pure organic solvents, several polarity parameters based on equilibrium, kinetic and spectroscopic measurements are discussed in detail by Reichardt and Welton (2011) [44]. Probably the most familiar approach to describe both the bulk solvent and the surface properties of various solid materials, e.g., synthetic polymers, native polymers, inorganic oxides, sol-gel hybrids, and composites is through the parameters of the Kamlet-Taft equation, expressed in a simple mode as:

CP = CP0 + sπ* + aα + bβ(1)

where CP is the property to be determined with reference to a standard system CP0, (i.e., gas phase or a non-polar solvent). Frequently, CP0 is the maximum frequency of absorption or emission in the gas phase while CP is the corresponding frequency in the presence of the solvent. From the viewpoint of solvent polarity, the most decisive parameter is π* (the measure of solvent dipolarity/polarizability), while β is related to the basicity of the solvent hydrogen bond acceptor, and α to the acidity of the solvent hydrogen bond donor). Coefficients s, a, and b are solvent independent indicating the respective influence of the π*, α, and β terms on the CP under study.

Measurements of choline chloride-based DESs with urea; glycerol; acetic acid; malonic acid; glycolic acid; ethylene glycol, or levulinic acid, as well as those of DESs composed of tetrabutylammonium chloride and levulinic acid indicate that their polarity is close to that of water [45,46]. The fact that the composition of two- or more-component DESs can be, in practice, varied nearly infinitely, opens a chance to prepare DESs with polarity fitting properties required by and suitable for desired applications. The values of parameters π*, α, and β for selected DESs are listed in Table 4.

The polarity of solvents may significantly affect the course of polymerization and cause alterations in polymer constitution, structure and behaviour. When dealing with polymers in solvents, the interaction takes place at the phase interface, the polymer being a solid component. Taking the polarity aspect into account, it is worth pointing out the ability of some solvents to exhibit switchable polarity [49]. Switchable polarity solvents (SPSs) equilibrate between a higher polarity and a lower polarity when a trigger is applied. These solvents are particularly useful when two different polarities of the solvent are needed for two different steps. SPSs have also penetrated into the field of polymer chemistry [49,50,51]. Up to now, mainly ionic liquids have been considered as belonging to the category of SPSs, there is, however, no fundamental reason hampering the introduction of DESs into the same category.

The polarity properties of polymers play an important role in practical industrial processes, such as dissolution of polymers in special solvents, adsorption of polymers on surfaces, and mixing of different polymers with each other. Mutual interactions of two or more different materials, e.g., the extent of the adsorption of flexible macromolecules to solid surfaces of inorganic or organic materials (particles, fibres, flat surfaces), the extent of the integration of inorganic fillers into organic polymer matrices, the catalytic activity of inorganic oxides etc., depend on the nature and strengths of the intermolecular forces of the components involved. Purposeful implementation of such interactions is conditioned, inter alia, by the knowledge of polarity of the interacting components. It was documented that the Kamlet-Taft equation may be applied, along with solvent properties, to describe the surface properties of various solid materials, e.g., synthetic polymers, native polymers, inorganic oxides, sol-gel hybrids, and composites using the Kamlet-Taft solvent parameters π*, α, and β as the reference system [41]. Thus, the polarity scale for solid materials was suggested. The values of π*, α, and β parameters for macromolecules can be determined using various solvatochromic polarity indicators a detalied report has been published by Reichardt and Welton (2011) [44]. The values of β, α, and π* parameters for selected solid materials are reproduced from [52] and listed in Table 5.

## 4. The Role of Deep Eutectic Solvents in the Synthesis of Polymers

In systems containing a DES, a monomer and a polymer, their interaction can lead to the following groups of consequences:

(1) The DES can function just as a solvent, without being involved directly in the conversion of monomer to polymer occurring in the system, however, influencing the course (e.g., kinetics) of the conversion;
(2) One of the DES components can itself undergo polymerization;
(3) The presence of the DES facilitates or causes changes in polymer properties (mainly surface modification of cellulose or nanocellulose, nanofibers) or in the production of nanocellulose (isolation of cellulose nanocrystals and cellulose fibres from lignocellulosic biomass or cellulose fibres) or in the pretreatment of other renewable polymers, such as chitin, chitosan, starch).

In general, solvents in the condensed phase are able to alter the reaction rate even by a few orders of magnitude. This is due to the fact that the molecular orbital energy of the reagents can be greatly influenced by the solvents [53]. The reactivity of a monomer in radical polymerization is usually higher in polar media than in non-polar ones. The solvent is selected so as to increase the solubility of the produced polymer chain. Moreover, the solvent facilitates heat dissipation in the reaction system. The used solvents are usually inert and are not involved in the reaction mechanism. In the case of applying DESs, the medium may act as a reactant as well.

Upon polymerization, chain transfer due to the solvent cannot be omitted. Owing to the influence of the solvent possessing a high transfer effect, radicals of the initiator or of the monomer can abstract hydrogen atoms. A radical formed from a solvent molecule causes termination of the polymerization reaction [53]. As a result, a polymer with a lower molecular weight is produced. Among classic organic solvents with high transfer effect, tetrahydrofuran, isopropanol, diisopropyl ether, chloroform and alkylated benzenes [54] can be mentioned. When polymerization occurs in a solvent with a low chain transfer rate (e.g., *tert*-butanol, water, benzene), a polymer with a high molecular weight is formed. Mitra et al. found that radical transfer reactions by way of hydrogen atom abstraction from the organic substrates by the OH^▪^ radicals were more favoured in aqueous media than in non-aqueous systems [55]. This information can be of key importance when working with eutectic systems containing water. In recent years, several research teams have overviewed the possibilities of cellulose treatment, cellulose modification, processing of nanocellulose fibres, nanocrystals and microcrystalline nanocellulose by DESs [56,57,58].

## 5. Research on Polymerization in Deep Eutectic Solvents

### 5.1. Role of Deep Eutectic Solvents in Polymerization Processes

In the field of synthesis and polymerization with DESs, several DESs have been investigated, and the results of their usability have been examined [23,59,60]. Frontal polymerization occurs when the monomer becomes a polymer in a localized reaction zone that propagates in an unstirred medium, through the coupling of thermal transport and the Arrhenius dependence of the reaction rate of an exothermic polymerization [61,62]. Frontal polymerization has been applied to form polymer nanocomposites, such as macroporous poly(acrylic acid)-carbon nanotube composites [63], xerogels [64], or hydrogels [65,66,67]. Frontal polymerization has been carried out in a pure monomer but it is possible to obtain fronts of highly reactive monomers in high-temperature boiling solvents including water, DMSO and DMF [62]. 

Besides regularly examined and discussed characteristics of DESs, specifically with regard to their involvement in polymer chemistry, their boiling point, which stands for their maximum usability limit, is of key importance. As pointed out by Marcus [68], the boiling point of DESs represents the upper limit for their usage. The known boiling points of selected DESs are tabulated in Table 6. In general, the boiling point has not yet been understood as a relevant feature, on the basis of which, the application of DESs for different processes would be chosen. However, this situation may change because the solvent and its essential characteristics (e.g., boiling point, polarity, etc.) may serve as a tool to control the choice of the solvent and its application in polymerization reactions. Moreover, the choice of solvents should be also made by taking into account their heat transfer properties.

### 5.2. Polymerizable Deep Eutectic Solvents

Itaconic acid (2-methylidenebutanedioic acid) is one of the basic building blocks obtained from biomass. That is why, some research groups use it in polymerization reactions in combination with DESs, where it serves as HBD. A number of publications have reported the use of itaconic acid, along with choline chloride or tetraethylammonium chloride, in free-radical polymerization [64,67,70,71,72].

The results of Bednarz’ research group have been described in detail in a recent review [23]. Table 7 summarizes basic information about the works where itaconic acid has been applied in the system as HBD. Bednarz et al. prepared mixtures in a 1:1, 2:3 or 1:2 molar ratio, however, the mixtures with higher itaconic acid content tended to crystallize, and the authors therefore continued to use 1:1 mixtures [65]. In this 2014 work, they reported that the initiator of the polymerization reaction is not soluble in the DES (choline chloride and itaconic acid). In order to carry out the polymerization reactions, it was thus necessary to add a small amount of water to the system. They described the employment of choline chloride and itaconic acid (1:1) with a minimum amount of water, and ammonium sulphate and N,N′-methylenebisacrylamide as initiator. In this system, the yield of polymer conversion was higher than 90%. In the case of applying only an aqueous medium without the DES, the conversion was of only 50%. They found that the DES can directly influence the rate constant of initiator decomposition, choline chloride can activate the decomposition of sulphate to radicals. In the works by Abbott et al. and Bednarz et al. the authors claimed that quaternary ammonium salts can associate with protic substances via hydrogen bonding, and carboxylic monomer could be more reactive than nano-associated species [1,65].

In 2015, the Bednarz’ group concentrated on the mechanism of persulphate decomposition. They found that the presence of choline chloride increased the solubility of itaconic acid in water and supported the formation of poly(itaconic acid) with higher molecular weight and higher polydispersity [70]. They also confirmed the information that the decomposition of persulphates is important for the kinetics of the initiation step of free-radical polymerization in the presence of a small amount of water in the DES. All polymers were obtained with a yield of 50‒62%, molecular weight of Mw 1400–46000 g·mol^−1^, and polydispersity of 1.8–10.5.

The formation of macroporous itaconic xerogels has been described in [64]. The authors used the system composed of choline chloride, itaconic acid, ammonium persulphate and different amounts of crosslinking agent (2, 5, 10, 15 mol %), as well as other systems containing additional inert diluent poly(ethylene glycol) with different molar weight M = 1500 g·mol^−1^ and 3000 g·mol^−1^. As a result, the formation of xerogels, with the yield of the gel fraction of 29‒100% for the systems without poly(ethyleneglycol), and of 64, 74% for the systems containing poly(ethyleneglycol), was observed. The following year, Bednarz et al. reported on the formation of polymeric products at pre- (nanogel-like networks) and post-gel stages (macrogels) [72].

Their most recent paper describes the influence of ternary mixtures on the polymerization of itaconic acid [71]. In this study, they worked with the 1:1:1 system, composed of choline chloride or tetraethylammonium chloride, itaconic acid and water. Water was considered as the initiator solvent. When using choline chloride, persulphate initiated free-radical polymerization in ternary DES reached 96% conversion of the monomer, and the isolated yield of 79% (mw 34560, dispersity 3.6). In the system containing tetraethylammonium chloride, Mw of 14,320 g·mol^−1^ and dispersity of 2.6 were obtained (87% conversion of the monomer and isolated yield of 69%).

The most valuable conclusion of this work lies in understanding the impact of the solvent on the formed polymer structure. As a result, the presence of methylene and methine groups formed by decarboxylation and/or branching, as well as that of choline ester moieties, was identified in polymers. Non-polymerizable HBDs and polymerizable ammonium salts create deep eutectic monomers by free-radical polymerization, as presented for the first time in [73]. The authors prepared different DES monomers: 2-cholinium bromide methacrylate/citric acid; 2-cholinium bromide methacrylate/amidoxime methacrylate (photopolymerization was used to create polymers). On the other hand, polyesters were formed by polycondensation with the monomers: ammonium tetraol/ citric acid; ammoniumtriol/citric acid; ammonium tetraol/citric acid/terephthalic acid; ammonium triol/citric acid/terephthalic acid. Another work of Isik’s group was focused on the application of quick photopolymerization to obtain polymers [74]. Deep eutectic monomers were created based on cholinium bromide methacrylate with citric; oxalic; malonic; maleic acid; benzeneimidamide; trifluorobenzeimidamide; propanimidamide; diaminopyridine; piperazinediamine and diamino-propane.

### 5.3. Polymerization of Monomers by DESs

Polymerization of monomers by DESs described in the literature are listed in Table 8. Quin and Panzer used in situ UV copolymerization of 2-hydroxyethyl methacrylate (HEMA) and poly(ethylene glycol) diacrylate in choline chloride/ethylene glycol to create a new class of gel electrolytes [77]. A DES gel containing 13.2 vol% polymer scaffold was obtained.

In the DES composed of choline chloride and fructose, an ion gel was prepared by the self-polymerization of 2-hydroxyethylmethacrylate [78]. In the subsequent study the researchers investigated self-polymerization in the presence of choline chloride and orcinol [79]. As monomers, N-isopropyl acrylamide, vinyl acetate and 2-hydroxyethyl methacrylate (HEMA) were used. A highly stretchable gel was obtained only in the presence of HEMA [79].

A hydrated DES such as choline chloride with glycerol or urea was used for enzyme-mediated free radical polymerization of acrylamide [88]. The results of their experiments showed that the yield of polymer exceeded 90% for choline chloride/urea. and 99% for choline chloride/glycerol. Working with a system containing glycerol, a polymer with a higher molecular weight (Mw 9.85 × 10^4^ g·mol^-1^ (room temperature (RT)), 6.96 × 10^4^ g·mol^−1^ (50 °C)) and polydispersity (4.3; 8.3) than those of the polymer obtained using urea (Mw 9.85 × 10^4^ g·mol^−1^ (RT), 6.96 × 10^4^ g·mol^−1^ (50 °C)) and polydispersity (2.1; 2.8) was formed.

Wang et al. have explored the possibilities of employing atom transfer radical polymerization (ATRP) of methyl methacrylate (MMA) with FeBr_2_ in three types of DESs using totally 14 individual DESs under various conditions: FeBr_2_-catalyzed ATRP of MMA using different amide complexes; in caprolactam/acetamide solvent without additional ligands; in a trace amount of thiocyanate/amide DES without additives; and with a catalytic amount of inorganic base, Na_2_CO_3_; different types of quaternary ammonium salts with HBDs and with Na_2_CO_3_ [80]. The results of the work showed that by selecting the DESs and the conditions appropriately, functional polymers with different molecular weights (2400 to 74000 g·mol^−1^) and polydispersity (1.05 to 5.45) can be produced. The highest conversion, reaching 86.0%, was achieved using the DES composed of urea and NH_4_SCN under the following conditions: [MMA]0/[FeBr_2_]0/[EBPA]0/[Na_2_CO_3_]0 = 200:1:1:2; MMA/DES (*v*/*v*) = 200:1, 60 °C.

Nowadays, research focuses on the preparation of biodegradable polymers, one of them being polycaprolactone. Garcia-Arguelles et al. studied the option of ring-opening polymerization of ε-caprolactone in the presence of the DES composed of 1,5,7-triazabicyclo[4.4.0]dec-5-ene and methanesulfonic acid [81]. The characteristics of the formed polymer were as follows: degree of polymerization of 42–57, number-average molecular weight of 6120–9925 g·mol^−1^, and dispersity of 1.5–1.6. The reaction was run at a low temperature (37°C) and the reaction time was 2 h. The yield obtained under all the conditions used in the study was over 98% when the DES was used.

The influence of DESs on the formation of biodegradable polyesters, such as poly(octanediol-co-citrate) (POC), from a prepolymer with citric acid was investigated to obtain cross-linked polymers [82]. In this way, five polymers were created with the following molecular weights: 11,264 g·mol^−1^ (POC with choline chloride), 10,384 g·mol^−1^ (POC with tetraethylammonium bromide), 9,420 g·mol^−1^ (POC with hexadecyltrimethylammonium bromide), 14,970 g·mol^−1^ (POC with methyltriphenyl-phosphonium bromide (0.75), and 22,750 g·mol^−1^ (POC with methyltriphenylphosphonium bromide). The elastomer obtained from the DES containing bromide methyltriphenylphosphonium presents interest from the viewpoint of antibacterial activity, cytocompatibility and mechanical properties.

Serano and his coworkers prepared poly(diol co-citrate) elastomers in DESs consisting of lidocaine and 1.8-octanediol in different molar ratios, which subsequently reacted with citric acid [83].

Sapir et al. explored the effect of choline chloride/urea (1:2) DES on the properties of polyvinylpyrrolidine [93]. When comparing the properties using water and the DES, they found that the DES caused changes in the polymer structure and its properties (a higher degree of polymer swelling). The authors documented that the intermolecular interaction between polyvinyl pyrrolidine and choline chloride/urea is different from that with the water system, and the polymer formed in the DES maintained a flexible coil structure. Another application of DESs was studied in [94]. Electrospinning combined with the encapsulation of a DES (choline chloride/citric acid (1:1)) may be applied to obtain fibres. Electrospun fibres were smooth and uniform.

A recent work on synthetic recombinamers has demonstrated that the elastin-like polymers may be stabilized in their collapsed state due to the presence of DESs, particularly of hydrated DESs [95]. The ionic strength of the DES helped to stabilize elastin-like recombinamers in the collapsed state and contributed to the subsequent formation of aggregates upon the loss of the structural water molecules involved in hydrophobic hydration. In this work choline chloride: urea (1:2) was used.

Recently researchers have focused their attention on the DES-assisted polycondensation reactions of resorcinol-formaldehyde. The result was the formation of a carbon-carbon nanotube composite, with improved functionality. In the polymer phase, resorcinol or hexylresorcinol acts as precursor and HBA is segregated into the polymer depleted phase [59,96,97]. The properties of the carbon monoliths are influenced by the type of the DES and their HBA and HBD components [97].

## 6. Deep Eutectic Solvents for Isolation of Nanocellulose

### 6.1. Modes of Cellulose Isolation from Plant-Based Biomass

In the last five years several research teams have published data focused on the purposeful pretreatment, modification, and dissolution of cellulose from the following sources: cotton linter pulp [98,99,100,101]; microcrystalline cellulose [100,101]; hardwood or softwood pulp [102,103,104,105,106,107,108,109,110,111,112]; Loblolly pine particles [113]; cellulose methyl carbamate [114]; recycled boxboard, milk containerboard, fluting board [103,109,115]. Cellulose nanocrystals or cellulose whiskers, and cellulose nanofibrils are species, which one dimension must be less than 100 nm. Chemical hydrolysis of para-crystalline regions of cellulose produces cellulose nanocrystals from cellulose fibers. Mechanical treatment of cellulose fibers has been performed mainly by equipment such as homogenizer, microfluidizer, grinder, extruder, blender and by processes such as ultrasonication, cryocrushing, steam explosion, ball milling, and aqueous counter collision [27,28,29].

Cellulose plays an important role in the group of natural macromolecular substances. It is the most widespread natural polymer on earth and, moreover, it is an integral part of green biomass. Here, depending on where it is located and how it is arranged, it allows fulfilling various functions (biological, physicochemical, mechanical). The development of methods of isolation, purification and modification increases the intensification and rationalization of its industrial processing in various fields of industry (pharmacy, food industry, construction, etc.). In nature, we can also find it in a relatively clean state in some types of plants, cotton fibers, or plant lumps such as flax, hemp, ramia. However, it is usually present in the cell wall of each plant along with other substances, especially lignin, hemicelluloses, and pectin.

Cellulose contains hydroxyl groups. Based on its structure, cellulose reacts on the surface of macromolecules, on the surface of micelles, between micelles or even inside the micelle. Based on the complexity of the structure, the orientation of the micelles, the availability of the surface and the degree of swelling, various modes of cellulose reaction may happen [116]. Cellulose subjected to a DES does not swell, is highly oriented and, as a result, the reactions only occur on the surface. On the other hand, different kind of DES may cause that cellulose is unoriented or mercerized, and if DES do not cause its swelling, the reactions take place predominantly on the surface, however, the agent also partially penetrates into the micellar spaces. The cellulose dissolves at the reaction or it is already in dissolved form. The reaction may occur on the surface of micelles and macromolecules.

In the following part of this review we will try to summarize the possibilities and research activities in the following three basic areas: cellulose nanocrystals (Section 6.1); cellulose nanofibrils (6.2); and modification of cellulose (6.3).

### 6.2. Cellulose Nanocrystals

Liu and his coworkers demonstrated the effect of the eutectic mixture choline chloride/oxalic acid dehydrate (1:1) on cotton and its ability to produce CNCs in high yield [117]. Microwave irradiation was applied during the DES treatment process (power 800 W and temperature 70–100 °C). The yield of CFs from Gossypium hirstum L. (cotton fibers) was gradually reduced by heat stress from 74.2% (80 °C), through 62.4% (90 °C) to 57.8% (100 °C) after 3 min of microwave irradiation. Pretreated CFs were subsequently exposed to ultrasonication at 1200 W for 30 min. As a result, CNCs with different properties were formed. Due to ultrasonication the degree of polymerization of CFSs decreases from 1600 DP to 149, 125, and 109, based on different pretreatment temperatures. Based on morphological evaluation, the pretreatment temperature had an effect on dimensional characteristics. At 80 °C DES, the CF diameter was in a range of 3‒25 nm, and a length of 100‒350 nm. At 100 °C DES, the ultrasonication exhibits a more pronounced effect, shortening the diameter to less than 15 nm, and the length to 300 nm, mainly represented by a fraction of 100‒200 nm. Relative crystallinity index CrI reached 82–80.7% and compared to the original sample without ultrasonication (64.9%). The system’s crystallinity increased by 25%.

Ling et al. modified cotton fibers (*Gossypium hirstum* L.) using choline chloride/oxalic acid dihydrate in various molar ratios (1: 1; 1: 2; and 1: 3) at 80 °C or 100 °C, and 1:100 hydro modulus [118]. Subsequently, the treated fibers were subjected to high-power ultrasonic homogenization (300 W, 20 kHz, 1 h). As a result, CNCs were obtained with different dimensional characteristics, obeying the trend the higher molar ratio, the smaller CNCs dimensions: length (nm)/width (nm) at 80 °C (205.9/8.63; 171.9/7.40; 162/6.42); at 100 °C (194.1/9.62; 152.7/6.15; 122.4/4.69). Crystallinity index for treated samples at 80 °C were 75.27%; 73.85% and 70.81%; and for 100 °C 74.98%; 75.29% and 67.29%. In comparison with initial fibers (93.37%), the treatment by DESs led to a reduction of crystallinity. Important information and summarisation of results is interpretation effect of oxalic acid on the esterification of hydroxyl groups in C6 positions, and breakdown hydrogen bonding between the microfibrils. And this impact is connected with the more fibrillation of treated cotton fibers by application of system choline chloride and oxalic acid. On the other hand, the influence of acidity, i.e., higher oxalic acid concentration, is important because the formation of better dispersions and create shorter for length and width of CNCs. The higher thermal stress enhances this effect on the dimension characteristic.

Laitinen et al. focused their attention on stabilization of marine diesel oil in water emulsions by CNCs [115]. In this study the properties and function of two commercial nanocrystals and CNC synthetized from choline chloride and oxalic acid dehydrate were compared. Softwood pulp was pretreated with DES (6 h) and subsequently DES cellulose (pH 7, adjusted NaOH) was converted into nanocrystals (width 3 to 8 nm; length 50 to 350 nm, CrI 68%) by microfluidizer equipment. The efficiency of dispersant type of DES was comparable to that of commercial CNCs for 0.1 wt% dosage of dispersant.

Assessing the application of an acidic deep eutectic solvent consisting of choline chloride and oxalic acid (anhydrous); levulinic acid (1:2); dihydrate oxalic acid; and para-toluenesulfonic acid monohydrate (1:1) and the consequent mechanical treatment on the formation of nanocrystalline cellulose structures was studied in [107]. Due to the effect of acid pretreatment, the cellulose was degraded and its viscosity dropped from an initial value of 503 cm^3^·g to approx. 105 cm^3^.g (for levulinic acid 267 cm^3^.g). For samples pretreated by oxalic acid different an time of reaction (2–6 h), and temperature (100 and 120°C) were used. These samples were studied by X-ray diffraction after the disintegration. Tested samples demonstrated CrI values ranging from 66‒71%, width 9.9 nm to 15.7 nm, and length 337 to 390 nm. The CrI of treated samples was slightly higher than that in the original pulp (66%).

To dissolve the pulp or microcrystalline cellulose, in [119] a system consisting of (anhydrous) phosphoric acid and guanidine hydrochloride (2:1) was applied. After the dissolution (time 24 h, hydro modulus (1:20)) desintegrated cellulose was regenerated by a microfluidizer (1 min), and the dispersion passed through chambers (400, 200, 87 µm) several times at different pressures. The strong acid ion of the DES causes significant degradation of cellulose, after regeneration DP was 80 (initial DP of pulp 1800, microcrystalline cellulose 373) and CrI was around 80%.

Oxalic acid dihydrate with choline chloride and FeCl_3_ with different mass ratios (1:1, 2:1, 3:1, 4:1) and different charges of FeCl_3_ × 6H_2_O was applied to achieve CNCs with different properties (size, zeta potential, content of carboxylic groups) [120]. The optimal conditions for production of CNCs were 80 °C, 6 h, and under DES treatment with oxalic acid dihydrate/choline chloride/FeCl_3_ × 6H_2_O (4:1:0.2 (mass ratio)).

### 6.3. Cellulose Nanofibrils

The preparation of cellulose nanofibrils is a process in which cellulosic fibers are broken down in particular by mechanical force with subsequent aggregation of the formed nanofibrils. The forces that assist this process of high-pressure homogenization and cause these changes are related to the high pressure, change of pressure, shear, speed and turbulence generated during the homogenization. However, by subjecting the material to these forces, high input costs should be expected. This means that these processes are highly energy-intensive. Thus, the fibre conditioning/ treating devices are used, such as homogenizers, microfluidizers, grinders, extruders, blenders and cryocrushingball milling, and aqueous counter collision [27,28,29] usually require looking for ways to reduce the energy demands. The solution to this situation lies in the application of methods that primarily ensure the change in the behaviour of the raw material, such as hydrolysis (i.e., enzyme or acid), oxidation (TEMPO), carboxymethylation, quarterisation, alkylation, silanization, glyoxalization, esterification, and other chemical pretreatments [121]. The aim is to increase the grinding capability leading to softer fibers, reduce of energy demands of the grinding process, and improve the aggregation and stability of the resulting product [27,121]. One of such modern pathways is the use and application of green solvents to produce CNF in conjunction with the homogenization process using high-pressure homogenization, ultrasonication and microfluidization and grinding [104,108,114].

Birch cellulose pulp was pretreated by choline chloride/urea (1:2) and disintegration was performed in three degrees by a microfluidizer [98]. The mechanical process produced nanofibril bundles (width 15–200 nm) and CNFs widths ranging from 2–5 nm. An interesting result of this work is the finding that the DES used does not cause any change in the degree of polymerization in the initial pulp. Three different samples were prepared: NFC1 was passed three times through 400 μm and 200 μm chambers at a pressure of 1300 bar; NFC2 was passed additionally to NFC1 two times through 400 μm and 100 μm chambers at a pressure of 2000 bar; NFC3 passed additionally to NFC2 one time through 200 μm and 87 μm chambers at a pressure of 2000 bar. The crystallinity values after pulp disintegration were 63.2% (NFC1), 56.8% (NFC2), and 40.7% (NFC3), for the above mentioned different mechanical treatment. Initial CrI for pulp was 66%, and the DES does not affect the crystalline structure.

Li and his team focused on investigating the effect of two types of DES, consisting of ammonium thiocyanate/urea (1:2) and guanidine hydrochloride/urea (1:2) [104]. The pretreatment procedure was performed for 2 h, at 100 °C, treating 4 g of bleached birch (from *Betula pendula*) kraft paper and 400 g DES. A slight decrease in DP by approximately 5% was observed after pretreatment causing hemicellulose degradation in DES. In addition, there has been an increase of lateral dimension due to fibre swelling and loosening of the cell wall structure. After disintegration CNFs with different lateral dimensions (widths from 13 to 19.3 nm) were obtained. Regarding the mechanical characteristics of nanofibril films using guanidine hydrochloride/urea DES, tensile strength achieved 135 to 163 MPa, and elastic modulus 6.4 to 7.0 GPa. Pretreatment with ammonium thiocyanate/urea led to tensile strength up to 189 MPa and elastic modulus up to 7.7 GPa.

In their further work, authors focused on the production of cationic nanocelluloses by recyclable DES (aminoguanidine hydrochloride/glycerol) [122]. Obtained CNFs and CNCs achieved width 4.6 nm and 5.7 nm, respectively. In this study, they also investigated recyclation of the used DES and confirmed its effectiveness as a cationization medium. After a simple method of regeneration by distillation, the DES was reused five times, and the results indicated the potential of recyclable medium use on the formation of cationic nanocellulose.

The raw material such as recycled box board, recycled milk-container board, fluting board, and bleached birch (*Betula verrucosa*) kraft pulp were converted into porous, hydrophobic and superabsorbent CNF through pretreatment by DES (choline chloride/urea), nanofibrilation, silylation and freeze drying [102]. Based on the results obtained, the authors assessed the potential of prepared aerogels to absorb oils and organic compounds (marine diesel, kerosene, gasoline, castor, linseed motor oil, chloroform, hexane, DMSO, toluene, acetone and ethanol). CNF aerogels absorption capacities for various oils and solvents were in the range from 65 to 205 g.g^−1^ for all the aerogels. On the basis of the calculation of the cost of individual operations and material prices also the price of acquired aerogels was reported. The price of the aerogel from recycled board and for the aerogel from virgin pulp were estimated to be 3.8 EUR·kg^−1^ and 4.4 EUR·kg^−1^, respectively. Data published in [103] documented the reduction of crystallinity due to the through grinder. Fibers pretreated by DES without Masuko treatment had crystallinity 61‒66%, and after 10 passes through grinder the crystallinity decreased from 56 to 53%. Thus CNFs have a high crystalline structure.

The ramie fibers were disintegrated by ball milling, the fibers being pretreated using choline chloride-based DESs with urea (1:1) and oxalic acid dihydrate (1:2) [123]. They found that the system contained oxalic acid is more suitable for obtaining CNFs. The pretreatment effect facilitates the recovery of CNFs (CrI 66.51%, thermal stability 322.6 °C and tensile strength of the film 52 MPa), which is mainly due to the degradation effect of the acid on the cellulose (degree of polymerization: CNFs 305 again 782 for untreated CNFs).

System DES urea and LiCl (5:1) was used to succinylate cellulose pulp [106] under different temperature (70, 80, 90 and 100 °C) and time (2, 6, 24 h) conditions. This was followed by a selection of samples which were subsequently used in the consequent mechanical treatment by microfluidizer. Selection of samples was made on the basis of higher content of carboxyl groups (sample with 2h pretreatment were selected). Microfluidization caused a drop in CNF crystallinity ranging from 51.5 to 58.3%.

Water-containing DES (choline chloride/oxalic acid dihydrate, 1:1) was used to pretreat the kraft pulp, followed by ultrasonic treatment. This ultrasonic application did not have as much nanofibrillation efficiency, and pretreatment of specimens by DES has a better effect on CNF and CNC production [124]. Significant changes in crystallinity (initial pulp 65.88%, after DES treatment 69‒70%) were not confirmed. The using ultrasonic treatment (800 W, 20 min) allowed to obtain CNCs/CNFs with width 2‒50 nm and length >500 nm.

Biohybrid film with cationic CNFs and tannin extract was prepared, and its properties such as UV-shielding and antioxidant were evaluated [125]. The cationization of birch pulp was reached using periodate oxidation (LiCl + NaIO_4_) and the DES consists of aminoguanidine hydrochloride and glycerol (1:2). Dimension of CNFs was 4.6 nm and several hundreds of nm.

### 6.4. Modification of Cellulose

In particular, as mentioned above, the initiation of the cellulose reaction is conditioned by the accessibility of the hydroxyl groups in the macromolecule structure, or, more precisely, on the individual surface levels (fibre surface, cell wall layers, macrofibrils, microfibrils, elemental fibrils, cellulose macromolecules). The reactions take place on a geometric surface or in situ at different levels of the structure. Application of various types of reagents results in a change in the chemical composition and the emergence of new functional products. Some functional groups may change too, but the cellulose exposed to these agents in fibrous form may also contain unchanged molecules since the agent has not acted in its entire volume. In the 21st. century different types of green solvents, such as DESs, have appeared which may be used to modify cellulose; and open new ways to obtain functional products with unique properties. Utilization of green solvents is directed to the use of reactive DES to rearranged cellulose by nanofibrillation to obtain CNFs and CNCs.

Despite the fact that the results in several works suggest that cellulose solubility in most of these solvents is less than 5%, or that these systems do not dissolve cellulose at all, DES causes changes in the properties of cellulose, nanocrystals, and nanofibers [2,11,39,108,109,110,111,126].

The team headed by Ren showed that imidazole, urea, acetamide, ammonium thiocyanate and caprolactam in combination with choline chloride can be used to dissolve cellulose from cotton linter pulp [99]. In their following work, cotton linter was dissolved by the choline chloride/oxalic acid system, as well as by allyltrimethylammonium chloride/oxalic acid [98]. The highest solubility of cellulose (6.48%) was achieved at 110 °C. A number of analyses have provided proof of the bonding of the choline chloride nitrogen atom to cellulose and simultaneous interaction of the choline chloride cation with the anionic part of cellulose. One of the frequently used hydrogen bond donors in DES systems is urea. This is due to the fact that its effect on the interaction with hydroxyl groups in cellulose by forming cellulose carbamate is confirmed [111,112].

Abbott’s teams demonstrated that quarternary ammonium groups bonded to cellulose can be formed [2]. Through the interaction of choline chloride and urea a highly viscose gel-like suspension can be prepared [127]. Based on the characterization of its properties it was found that the effect of DES treatment did not significantly change the degree of polymerization and it did not cause changes in the crystalline structure and chemical composition.

In their follow-up work, the same authors attempted to explain the interactions between cellulose fibers and choline chloride and urea [111]. Several analyses have confirmed that there is a binding of the nitrogen on the cellulose tightly bound choline chloride, making the cationic choline ions by electrostatic interacting with the anionic group in cellulose. Syntheses of cellulose carbamate were performed by urea-based DESs containing choline chloride (2:1; 4:1); betaine (2:1; 4:1); betaine hydrochloride (2:1; 4:1) [112].

It was attempted to reach the direct change in cellulose properties using sulfamic acid and urea in different molar ratios (1:2, 1:3, and 1:4). Simultaneously, the sulfamic acid to cellulose ratio was changed from 10: 1, through 6:1, to 3:1 [119]. As a result, DES caused a decrease of DP by up to 17–46% (initial DP of pulp 1822). They found that a higher strong acid content had a more pronounced effect on cellulose degradation, hence the addition of urea decreases cellulose degradation. Content of sulphate group increases with the amount of acid, surprising is the increase that with the reduction of molar ratio between substrate and acid increases cellulose degradation.

## 7. Polymers from Renewable Sources: Future Perspectives

In recent years, many researchers have used DESs in organic chemistry [128,129], but nowadays DESs have found their way also into research in the polymer industries, particularly in polymerization and synthesis of polymers and various composite types. The possibilities of using these solvents are not yet fully explored, but work is underway to investigate their applications. Through the combination of different types of HBA and HBD we can get DESs with different properties that affect the resulting polymerization process (reactivity, diffusion coefficients and other factors). Such an approach may allow the properties of the systems be tuned to achieve desirable courses in polymerization processes and quality of their products.

As it is well-known in organic chemistry and synthesis, solvent polarity significantly affects the course of organic reactions. There is no justified reason to also not take into account the effect of polarity when dealing with the course of polymerization processes. These effects are not yet understood in detail and clearly explained in polymer chemistry when applying DESs. It can be expected that investigation of these aspects will be of interest to both theorists and technologists working in polymer chemistry.

In area of DESs applications, systems are often used with water and some of them are hygroscopic. Based on the structure of the DES and NADES components, it is necessary to clarify the influence of water both on these systems and the polymerization reactions. In addition to the processes of direct preparation of polymers or composites themselves, the field of application of DESs for the modification of existing polymers is incredible wide. By the appropriate choice of DES system, the functional properties of the polymers can be altered. In the case of periodic frontal polymerization it would be appropriate to examine single and multi-point spin modes in which local high-temperature regions migrate around the front as the monomer propagates. It would also be useful to determine the impact of initial temperature and heat loss.

Near future will require exploring the possibility of DESs application as solvents for controlled radical polymerization, and attempt to understand associated structure of eutectic systems and interactions linked to the reagents. The following step will be to describe the mechanisms of polymer formation and explain the role of the HBA and HBD during the reaction.

Despite the fact that the role of DESs in the polymer industries is still largely unexplored, a rapid increase in research and implementation of results can be expected. In particular, the use of these solvents in the production of biodegradable plastics can, to a considerable extent, expand their use as a new breakthrough technology in greener polymer industries.

Another important area of industrial application and utilization of green solvents covers cellulose nanomaterials or nanocomposites. The objective is the preparation of CNCs or CNFs with the use of shearing equipment proceeding by the processing of feedstock in order to change accessibility and improve the release of individual microfibrils. In addition, the field of cellulose modifying is currently flourishing with the aim of obtaining suitable properties for further use in various industries: biopolymers, paper, coatings, flotation, food, pharmaceuticals, and textiles. The utilization of DESs for chemically treated cellulose creates a new plan for the sustainable modernization of cellulose use. Potential applications of nanocellulose or functionalized polymer matrices lie in surface sizing and coating processing as a barrier against vapour or gases, introducing lysols, suspensions, emulsions and foams, hydrogels and food thickeners into food packaging.

## Figures and Tables

**Table 1:** Classification of deep eutectic solvents [34].

Type	General formula	Terms
I	Cat^+^X^−^ × z MCl_x_ (quarternary halide + metal chloride)	M = Zn^II^, Sn^II^, Al^III^, Ga^III^, In^III^; X = Cl, Br
II	Cat^+^X^−^ × z MCl_x_ × y H_2_O (quarternary halide + metal chloride hydrate)	M = Cr^III^, Co^II^, Cu^II^, Fe^II^, Ni^II^; X = Cl, Br
III	Cat^+^X^−^ × z RZ (quarternary halide + hydrogen bond donor)	Z = CONH_2_, COOH, OH; X = Cl, Br
IV	MCl_x_ + RZ = (MCl_x−1_)^+^ × RZ + (MCl_x+1_)^−^ (metal halide hydrate + hydrogen bond donor	M = Al^III^, Zn^II^; Z = CONH_2_, OH

**Table 2:** Composition and physical properties of selected two-component Class III DESs at 298 K (if not specified otherwise); data extracted from [11].

HBA/HBD, Molar Ratio	Freezing Point [°C]	Viscosity [cP]	Density [g cm^−3^]	Conductivity [mS cm^−1^]
ChCl/U, 1:2	12	750	1.25	0.75
ChCl/U, 1:2	12	169 (40 °C)		0.199 (40 °C)
ChCl/Me-U, 1:2	29			
ChCl/1,3-Me_2_-U, 1:2	70			
ChCl/CF_3_CONH_2,_ 1:2		77 (40 °C)	1.342	
ChCl/EG, 1:2	−20	37	1.12	7.61
ChCl/EG, 1:3		19 (20 °C)		
ChCl/Gly, 1:2	−40	376	1.18	1.18
ChCl/Gly, 1:3		450 (20 °C)		
ChCl/D-Fru, 1:2	4			
ChCl/D-Glu, 1:2	14			
ChCl/phenol, 1:3			44.6	3.14
ChCl/phenol, 1:3			28.2 (35 °C)	4.77 (35 °C)
ChCl/phenol, 1:3			19.2 (45 °C)	6.77 (45 °C)
ChCl/1,4-butanediol, 1:3		140 (20 °C)		
ChCl/1,4-butanediol, 1:4		88 (20 °C)		
ChCl/imidazole, 3:7		15 (70 °C)		12 (60 °C)
ChCl/acetamide, 1:2	51			
ChCl/MalA, 1:1	10	721		0.55
ChCl/MalA, 1:2		1124		
ChCl/valeric acid, 1:2	22			
ChCl/mandelic acid, 1:2	33			
ChCl/OxA, 1:1	34			
ChCl/phenylacetic acid, 1:1	25			
ChCl/glutamic acid, 1:2	13			
ChCl/phenylpropionic acid, 1:1	20			
ChCl/CitA, 2:1	69			
[P(Me)(Ph)_3_]Br/Gly, 1:1.75	−4.03	887 (45 °C)	1.29	0.165
[P(Me)(Ph)_3_]Br/EG, 1:4	−49.34	109.8	1.23	0.788
[N(Bu)_4_]Br/imidazole, 3:7		810 (20 °C)		0.24 (60 °C)
[P(Bz)(Ph)_3_]Cl/EG	47.91			
[P(Bz)(Ph)_3_]Cl/Gly	50.36			
[NH_3_(Et)]Cl/acetamide, 2:3		64 (40 °C)	1.041	0.688 (40 °C)
[NH_3_(Et)]Cl/CF_3_CONH_2_, 2:3		256 (40 °C)	1.273	0.39 (40 °C)
[NH_3_(Et)]Cl/U, 2:3				0.348 (40 °C)
[N(Bu)_3_(Me)]Cl/EG, 1:3		202 (55 °C)		0.48 (55 °C)
[N(Bu)_3_(Me)]Cl/Gly, 1:5		534 (55 °C)		0.163 (55 °C)

CF_3_CONH_2_–2,2,2-Trifluoroacetamide, 1,3-Me2U–1,3-dimethylurea, CitA–Citric acid, ChCl–Choline chloride, EG–Ethylene glycol, Fru–Fructose, Glu–Glucose, Gly–Glycerol, MalA–Malonic acid, MeU–Methylurea, U–Urea, OxA–Oxalic acid, [P(Me)(Ph)_3_]Br–Methyltriphenylphosphonium bromide, P(Bz)(Ph)_3_]Cl–Benzoyltriphenylphosphonium chloride, [N(Bu)_4_]Br–Tetrabuthylammonium bromide, [NH_3_(Et)]Cl–Ethylammonium chloride, [NH_3_(Et)]Cl–Ethylammonium chloride, [N(Bu)_3_(Me)]Cl–Tributylmethylammonium chloride.

**Table 3:** Composition and physical properties of selected ternary Class III DESs at 298 K (AA = acetamide; [BMIM]Cl = 1-Butyl-3-methyl imidazolium chloride; U = urea; ChCl = choline chloride; Gly = glycerol; MA = malic acid; [BMPI]Br = 1-butyl-1-methylpyrrolidinium bromide); gt = glass transition temperature.

Composition, Molar Ratio.	Freezing Point [°C]	Viscosity [cP]	Density [g cm^−3^]	Ref.
ChCl/MA/Gly, 1:1:1			1.254	[37]
ChCl/MA/Gly, 2:1:3	−54^gt^		1.148	
ChCl/MA/Gly, 1:2:1	−52^gt^		1.263	
[BMPI]Br/ChCl/Gly, 2:1:2	−24.0^gt^	14.46		[38]
[BMPI]Br/ChCl/Gly, 1:1:2	−26.0^gt^	11.57		
[BMPI]Br/ChCl/Gly, 1:2.4	−24.5^gt^	14.81		
[BMPI]Br/ChCl/Gly, 1:5:10	−27.0^gt^	9.33		

**Table 4:** Kamlet-Taft parameters of water, organic compounds and DESs, in different molar ratios, using a set of probes: a (N,N-diethyl-4-nitroaniline, 4-nitroaniline and pyridine-N-oxide); b (Reichardt’s betaine dye 33, 4-nitroaniline, N,N-diethyl-4-nitroaniline) [45,46].

Composition, Molar Ratio	α	β	π*	Ref.
Water	1.23	0.49	1.14	[47]
Butanoic acid	1.06	0.22	0.47	[46]
Hexanoic acid	1.05	0.21	0.43	[46]
Octanoic acid	0.94	0.23	0.38	[46]
Methanol	0.93	0.66	0.58	[46]
Ethanol	0.83	0.75	0.51	[46]
Glycerol	1.21	0.51	0.62	[46]
Choline chloride/Glycerol, 1:2	0.937	0.544	1.61	[48]
[N(Et)_4_]Cl/Butanoic acid, 1:2	0.99	0.76	0.92	[46]
[N(Et)_4_]Cl/Hexanoic acid, 1:2	0.97	0.85	0.86	[46]
[N(Et)_4_]Cl/Octanoic acid, 1:2	0.96	0.87	0.81	[46]
[N(Pr)_4_]Cl/Butanoic acid, 1:2	0.94	0.84	0.93	[46]
[N(Pr)_4_]Cl/Hexanoic acid, 1:2	0.91	0.92	0.85	[46]
[N(Pr)_4_]Cl/Octanoic acid, 1:2	0.90	0.96	0.80	[46]
[N(Bu)_4_]Cl/Butanoic acid, 1:2	0.92	0.99	0.86	[46]
[N(Bu)4]Cl/Hexanoic acid, 1:2	0.90	1.02	0.81	[46]
[N(Bu)_4_]Cl/Octanoic acid, 1:2	0.84	1.19	0.80	[46]
[N(Bu)_4_]Cl/Decanoic acid, 1:2	0.85	1.28	0.69	[46]
[N(Bu)_4_]Cl/Decanoic acid, 1:1	0.91	1.21	0.86	[46]
[N(Pr)_4_]Br/Butanoic acid, 1:2	1.07	0.80	0.93	[46]
[N(Pr)_4_]Br/Hexanoic acid, 1:2	1.02	0.86	0.87	[46]
[N(Bu)_4_]Br/Butanoic acid, 1:2	1.02	0.81	0.93	[46]
[N(Bu)_4_]Br/Butanoic acid, 1:1	1.09	0.84	0.90	[46]
[N(Bu)_4_]Br/Butanoic acid, 2:1	0.94	0.82	0.95	[46]
[N(Bu)_4_]Br/Hexanoic acid, 1:2	1.02	0.93	0.92	[46]
[N(Bu)_4_]Br/Octanoic acid, 1:2	0.98	1.09	0.84	[46]
[N(Bu)_4_]Br/Decanoic acid, 1:2	0.95	1.05	0.71	[46]
Choline chloride/Acetic acid, 1:2		0.53	1.10	[45]
Choline chloride /Levulinic acid, 1:2	0.51	0.57	1.00	[45]
Choline chloride /Malonic acid, 1:1	1.39	0.42	1.08	[45]
Choline chloride /Glycolic acid, 1:1	0.49	0.50	1.08	[45]
Choline chloride /Urea, 1:2	1.42	0.50	1.14	[45]
Choline chloride /Ethylene glycol, 1:2	1.47	0.57	1.07	[45]
Choline chloride /Glycerol, 1:2	1.49	0.52	1.11	[45]
DL-Menthol/Acetic acid, 1:1	1.64	0.60	0.53	[45]
DL-Menthol/Levulinic acid, 1:1	1.56	0.58	0.66	[45]
DL-Menthol/Octanoic acid, 1:1	1.77	0.50	0.41	[45]
DL-Menthol/Dodecanoic acid, 2:1	1.79	0.57	0.37	[45]
[N(Bu)_4_]Cl/Levulinic acid, 1:2		0.82	1.06	[45]
[N(Bu)_4_]Cl/Octanoic acid, 1:2	1.41	0.99	0.76	[45]
[N(Bu)_4_]Cl/Decanoic acid, 1:2	1.36	0.97	0.73	[45]
[N(Bu)_4_]Cl/Dodecanoic acid, 1:2	1.45	1.04	0.71	[45]

**Table 5:** Values of Kamlet-Taft π*, α, and β parameters for solid materials [52].

Material	α	β	π*	Notes
Polystyrene	0.00	0.28	0.66	
Poly(ethylene oxide)	0.00	0.65	0.86	
Poly(methyl methacrylate)	0.00	0.38	0.71	
Poly(vinylpyrrolidone)	0.01	0.93	0.93	
Polyvinylchloride	0.33	0.04	0.16	
Poly(vinyl acetate)	0.00	0.70	0.77	
Never dry cellulose	0.98		0.66	
Chitin	0.67		0.91	
γ-Al_2_O_3_	1.44		0.59	dried at 673 K
γ-AlO(OH)	1.49		0.66	dried at 1 073 K
Silica (KG 60)	1.13		1.08	dried at 673 K
Aerosil 300^®^ untreated	1.17	−0.23	0.94	room temperature
Aerosil 300^®^ dried	1.06	−0.26	0.92	room temperature
LiChrospher^®^ 18-5 μm	1.04		0.83	surface coverage 0.81 μmol·m^–2^

**Table 6:** Boiling points [69].

Composition, Molar Ratio	Boiling Point (K)
Choline chloride/Urea, 1:2	445.6
Choline chloride/Ethylene glycol, 1:2	439.0
Choline chloride/Glycerol, 1:2	515.4
Choline chloride/Malonic acid, 1:2	550.3
Choline chloride/Butanediol, 1:3	471.0
Choline chloride/Trifluoroacetamide, 1:2	408.8
Choline chloride/Lactic acid, 10:13	495.2
Choline chloride/Phenol, 1:2	445.3
Acetylcholine chloride/Urea, 1:2	461.6
Ethylammonium chloride/Urea, 2:3	381.6
Ethylammonium chloride/Acetamide, 2:3	351.8
Ethylammonium chloride/Trifluoroacetamide, 2:3	348.5
N,N-diethylethanolammonium chloride/Ethylene glycol, 1:2	446.5
N,N-diethylethanolammonium chloride/Glycerol, 1:2	522.9
Methyltriphenylphosphonium bromide/Glycerol, 1:2	635.4
Methyltriphenylphosphonium bromide/Ethylene glycol, 1:3	526.7
Methyltriphenylphosphonium bromide/Triethylene glycol, 1:5	608.0
Choline chloride /Glycerol, 1:1	500.9
Choline chloride /Glycerol, 1:3	522.6
N,N-diethylethanolammonium chloride/Trifluoroacetamide, 1:2	416.4
Choline chloride /Ethylene glycol, 1:3	436.7
Choline chloride /Phenol, 1:3	443.8
Choline chloride /Phenol, 1:4	442.9
Choline chloride /Lactic acid, 2:3	497.5
Choline chloride /Lactic acid, 1:2	502.0
Choline chloride /Lactic acid, 2:5	505.2
Choline chloride /Lactic acid, 1:3	507.6
Choline chloride /Lactic acid, 2:7	509.4
Choline chloride /Lactic acid, 1:4	510.9
Choline chloride /Lactic acid, 1:5	513.1
Choline chloride /Lactic acid, 1:8	516.8
Choline chloride /Lactic acid, 1:10	518.2
Choline chloride /Lactic acid, 1:15	520.1
Methyltriphenylphosphonium bromide/Glycerol, 4:7	643.7
Methyltriphenylphosphonium bromide/Ethylene glycol, 1:4	507.3
Choline chloride /Fructose, 5:2	574.9
Choline chloride /Fructose, 2:1	594.5
Choline chloride /Fructose, 3:2	621.9
Choline chloride /Fructose, 1:1	663.0

**Table 7:** Polymerizable DESs.

Composition, Molar Ratio	Initiator	Conversion (%)	Ref.
Acrylic acid/Choline chloride, 5:8	1,1-bis(tert-butylperoxy)-3,3,5-trimethylcyclohexane (Luperox 231) (0.2 mol %)	100	[75]
Acrylic acid/Choline chloride, 5:8	Luperox 231 (0.3 mol %)	100	[75]
Acrylic acid/Choline chloride, 5:8	Luperox 231 (0.4 mol %)	94	[75]
Acrylic acid/Choline chloride, 5:8	Luperox 231 (0.5 mol %)	93.6	[75]
Acrylic acid/Choline chloride, 5:8	Luperox 231 (0.7 mol %)	94.2	[75]
Acrylic acid/Choline chloride, 5:8	Luperox 231 (1 mol %)	92.5	[75]
Methacrylic acid/Choline chloride, 1:2	Luperox 231 (0.2 mol %)	98	[75]
Methacrylic acid/Choline chloride, 1:2	Luperox 231 (1 mol %)	100	[75]
Acrylic acid/Lidocaine hydrochloride, 3:1	Luperox 231 (1.5 mol %)	100	[75]
Acrylic acid/Lidocaine hydrochloride, 3:1	Luperox 231 (0.2 mol %)	100	[75]
Acrylamide/Choline chloride, 1:2	Luperox 231 (0.1 mol %)	90	[75]
Acrylic acid/Choline chloride + acrylamide, 1:2	Luperox 231 (0.03 mol %)	100	[75]
Acrylic acid/Choline chloride + N-isopropylacrylamide, 1:2	Luperox 231 (0.03 mol %)	100	[75]
Choline chloride/Acrylic acid, 5:8; 1:2	Crosslinkers: poly(ethylene glycol) diacrylate and 2-hydroxy-4-(2-hydroxyethoxy)-2-methylpropiophenone photoinitiator		[76]
Choline chloride/Itaconic acid, 1:1	Initiator: N,N′-methylenedisacrylamide, ammonium sulphate	>90	[65]
Choline chloride/Itaconic acid, 1:1	Initiator: N,N′-methylenedisacrylamide (2–15 mol %, ammonium sulphate	29–100	[64]
Choline chloride/Itaconic acid, 1:1	Initiator: N,N′-methylenedisacrylamide, ammonium sulphate, second inert diluent–poly(ethyleneglycol)	64–74	[64]
Choline chloride/Itaconic acid, 1:1	Initiator: N,N′-methylenedisacrylamide (2-15 mol %, ammonium sulphate	90–99 (post-gel stage)	[72]
Choline chloride/Itaconic acid, 1:1	Initiator: N,N′-methylenedisacrylamide (2-15 mol %, ammonium sulphate	90–99 (pre-gelation stage)	[72]
Choline chloride/Itaconic acid/Water, 1:1:1	Initiator: ammonium sulphate	96	[71]
Tetraethylammonium chloride/Itaconic acid/Water, 1:1:1	Initiator: ammonium sulphate	87	[71]
Lidocaine hydrochloride/Acrylic acid, 1:3	Luperox 231, as thermal initiator and ethylene glycol methacrylate or pentaerithrytol triacrylate	100	[66]
Lidocaine hydrochloride/Methacrylic acid, 1:3	Luperox 231, as thermal initiator and ethylene glycol dimethacrylate or pentaerithrytol triacrylate	100	[66]
Lidocaine hydrochloride/Acrylic acid + Lidocaine hydrochloride/Methacrylic acid (1:1), mixture	Luperox 231, as thermal initiator and ethylene glycol dimethacrylate or pentaerithrytol triacrylate	100	[66]
2-Cholinium bromide methacrylate/Citric acid, 1:1	Photopolymerization, crosslinker ethylene glycol dimethacrylate, photoinitiator 2-hydroxy-2-methylpropiophenone		[73]
2-Cholinium bromide methacrylate /Amidoxime methacrylate, 1:1	Photopolymerization, crosslinker ethylene glycol dimethacrylate, photoinitiator 2-hydroxy-2-methylpropiophenone		[73]
Ammonium tetraol/citric acid, 3:4	Polycondensation	≈90	[73]
Ammoniumtriol/citric acid, 1:1	Polycondensation	≈90	[73]
Ammonium tetraol/citric acid/terephthalic acid, 6:5:4	Polycondensation	≈90	[73]
Ammonium triol/citric acid/terephthalic acid, 8:5:4	Polycondensation	≈90	[73]
Cholinium bromide methacrylate/Citric acid, 1:1	Photopolymerization, crosslinker ethylene glycol dimethacrylate, photoinitiator 2-hydroxy-2-methylpropiophenone	100	[74]
Cholinium bromide methacrylate/Citric acid, 2:3	Photopolymerization, crosslinker ethylene glycol dimethacrylate, photoinitiator 2-hydroxy-2-methylpropiophenone	100	[74]
Cholinium bromide methacrylate/Oxalic acid, 1:2	Photopolymerization, crosslinker ethylene glycol dimethacrylate, photoinitiator 2-hydroxy-2-methylpropiophenone	100	[74]
Cholinium bromide methacrylate/Malonic acid, 1:2	Photopolymerization, crosslinker ethylene glycol dimethacrylate, photoinitiator 2-hydroxy-2-methylpropiophenone	100	[74]
Cholinium bromide methacrylate/Maleic acid, 1:2	Photopolymerization, crosslinker ethylene glycol dimethacrylate, photoinitiator 2-hydroxy-2-methylpropiophenone	100	[74]
Cholinium bromide methacrylate/Benzeneimidamide, 1:1	Photopolymerization, crosslinker ethylene glycol dimethacrylate, photoinitiator 2-hydroxy-2-methylpropiophenone	100	[74]
Cholinium bromide methacrylate/Trifluorobenzeimidamide, 1:1	Photopolymerization, crosslinker ethylene glycol dimethacrylate, photoinitiator 2-hydroxy-2-methylpropiophenone	100	[74]
Cholinium bromide methacrylate/Propanimidamide, 1:1	Photopolymerization, crosslinker ethylene glycol dimethacrylate, photoinitiator 2-hydroxy-2-methylpropiophenone	100	[74]
Cholinium bromide methacrylate/Diaminopyridine, 1:1	Photopolymerization, crosslinker ethylene glycol dimethacrylate, photoinitiator 2-hydroxy-2-methylpropiophenone	100	[74]

**Table 8:** Polymerization of monomers in DESs.

Composition, Molar Ratio	Monomer	Agent	Time (h); Conversion (%)	Note	Ref.
Acetamide/Urea, 2:1	Methyl methacrylate	FeBr_2_	14.5; 9.6	[MMA]_0_/[FeBr_2_]_0_/[EBPA]_0_/[Na_2_CO_3_]_0_ = 200:1:1:0; MMA/DES (*v*/*v*) = 3:1, 60 °C	[80]
Acetamide/Urea, 2:1	Methyl methacrylate	FeBr_2_	6; 32.5	[MMA]_0_/[FeBr_2_]_0_/[EBPA]_0_/[Na_2_CO_3_]_0_ = 200:1:1:2; MMA/DES (*v*/*v*) = 200:1, 60 °C	[80]
Urea/Caprolactam, 1:3	Methyl methacrylate	FeBr_2_	14.5; 13.3	[MMA]_0_/[FeBr_2_]_0_/[EBPA]_0_/[Na_2_CO_3_]_0_ = 200:1:1:0; MMA/DES (*v*/*v*) = 3:1, 60 °C	[80]
Urea/Caprolactam, 1:3	Methyl methacrylate	FeBr_2_	6; 36.3	[MMA]_0_/[FeBr_2_]_0_/[EBPA]_0_/[Na_2_CO_3_]_0_ = 200:1:1:2; MMA/DES (*v*/*v*) = 200:1, 60 °C	[80]
Caprolactam/ Acetamide, 1:1	Methyl methacrylate	FeBr_2_	3.5; 18.1	[MMA]_0_/[FeBr_2_]_0_/[EBPA]_0_/[Na_2_CO_3_]_0_ = 200:1:1:0; MMA/DES (*v*/*v*) = 3:1, 60 °C	[80]
Caprolactam/ Acetamide, 1:1	Methyl methacrylate	FeBr_2_	5; 77.4	[MMA]_0_/[FeBr_2_]_0_/[EBPA]_0_/[Na_2_CO_3_]_0_ = 200:1:1:2; MMA/DES (*v*/*v*) = 200:1, 60 °C	[80]
Caprolactam/ Acetamide, 1:1	Methyl methacrylate	FeBr_2_	4.5; 78.8	[MMA]_0_/[FeBr_2_]_0_/[EBPA]_0_/[Na_2_CO_3_]_0_ =50:1:1:0; MMA/DES (*v*/*v*) = 3:1, 60 °C	[80]
Caprolactam/ Acetamide, 1:1	Methyl methacrylate	FeBr_2_	1.5; 76.5	[MMA]_0_/[FeBr_2_]_0_/[EBPA]_0_/[Na_2_CO_3_]_0_ =50:1:1:2; MMA/DES (*v*/*v*) = 3:1, 60 °C	[80]
Caprolactam/ Acetamide, 1:1	Methyl methacrylate	FeBr_2_	7.5; 46.4	[MMA]_0_/[FeBr_2_]_0_/[EBPA]_0_/[Na_2_CO_3_]_0_ =100:1:1:0; MMA/DES (*v*/*v*) = 3:1, 60 °C	[80]
Caprolactam/ Acetamide, 1:1	Methyl methacrylate	FeBr_2_	2; 58.6	[MMA]_0_/[FeBr_2_]_0_/[EBPA]_0_/[Na_2_CO_3_]_0_ = 100:1:1:2; MMA/DES (*v*/*v*) = 3:1, 60 °C	[80]
Caprolactam/ Acetamide, 1:1	Methyl methacrylate	FeBr_2_	3.5; 18.1	[MMA]_0_/[FeBr_2_]_0_/[EBPA]_0_/[Na_2_CO_3_]_0_ = 200:1:1:0; MMA/DES (*v*/*v*) = 3:1, 60 °C	[80]
Caprolactam/ Acetamide, 1:1	Methyl methacrylate	FeBr_2_	2; 28.5	[MMA]_0_/[FeBr_2_]_0_/[EBPA]_0_/[Na_2_CO_3_]_0_ = 200:1:1:2; MMA/DES (*v*/*v*) = 3:1, 60 °C	[80]
Acetamide/ NH_4_SCN, 3:1	Methyl methacrylate	FeBr_2_	18; 25.2	[MMA]_0_/[FeBr_2_]_0_/[EBPA]_0_ = 200:1:1; MMA/DES (*v*/*v*) = 200:1, 60 °C	[80]
Acetamide/ NH_4_SCN, 3:1	Methyl methacrylate	FeBr_2_	14; 42.0	[MMA]_0_/[FeBr_2_]_0_/[EBPA]_0_ = 100:1:1; MMA/DES (*v*/*v*) = 100:1, 60 °C	[80]
Urea/NH_4_SCN, 3:2	Methyl methacrylate	FeBr_2_	7; 13.0	[MMA]_0_/[FeBr_2_]_0_/[EBPA]_0_ = 200:1:1; MMA/DES (*v*/*v*) = 200:1, 60 °C	[80]
Urea/NH_4_SCN, 3:2	Methyl methacrylate	FeBr_2_	14; 37.0	[MMA]_0_/[FeBr_2_]_0_/[EBPA]_0_ = 200:1:1; MMA/DES (*v*/*v*) = 100:1, 60 °C	[80]
Acetamide/NH_4_SCN, 3:1	Methyl methacrylate	FeBr_2_	1.85; 84.7	[MMA]_0_/[FeBr_2_]_0_/[EBPA]_0_/[Na_2_CO_3_]_0_ = 200:1:1:2; MMA/DES (*v*/*v*) = 200:1, 60 °C	[80]
Urea/NH_4_SCN, 3:2	Methyl methacrylate	FeBr_2_	1.67; 86.0	[MMA]_0_/[FeBr_2_]_0_/[EBPA]_0_/[Na_2_CO_3_]_0_ = 200:1:1:2; MMA/DES (*v*/*v*) = 200:1, 60 °C	[80]
Caprolactam/ NH_4_SCN, 3:1	Methyl methacrylate	FeBr_2_	4.75; 74.3	[MMA]_0_/[FeBr_2_]_0_/[EBPA]_0_/[Na_2_CO_3_]_0_ = 200:1:1:2; MMA/DES (*v*/*v*) = 200:1, 60 °C	[80]
Acetamide/KSCN, 3:1	Methyl methacrylate	FeBr_2_	3.50; 70.2	[MMA]_0_/[FeBr_2_]_0_/[EBPA]_0_/[Na_2_CO_3_]_0_ = 200:1:1:2; MMA/DES (*v*/*v*) = 200:1, 60 °C	[80]
Caprolactam/ KSCN, 3:1	Methyl methacrylate	FeBr_2_	3.67; 80.5	[MMA]_0_/[FeBr_2_]_0_/[EBPA]_0_/[Na_2_CO_3_]_0_ = 200:1:1:2; MMA/DES (*v*/*v*) = 200:1	[80]
Choline chloride/Urea, 1:2	Methyl methacrylate	FeBr_2_	4.0; 70.6	[MMA]_0_/[FeBr_2_]_0_/[EBPA]_0_/[Na_2_CO_3_]_0_ = 200:1:1:2; MMA/DES (*v*/*v*) = 200:1, 60 °C	[80]
Choline chloride/ Ethylene glycol, 1:2	Methyl methacrylate	FeBr_2_	46.0; 14.4	[MMA]_0_/[FeBr_2_]_0_/[EBPA]_0_/[Na_2_CO_3_]_0_ = 200:1:1:2; MMA/DES (*v*/*v*) = 200:1, 60 °C	[80]
Choline chloride/Glycerol, 1:2	Methyl methacrylate	FeBr_2_	21.0; 21.5	[MMA]_0_/[FeBr_2_]_0_/[EBPA]_0_/[Na_2_CO_3_]_0_ = 200:1:1:2; MMA/DES (*v*/*v*) = 200:1	[80]
Choline chloride/Malonic acid, 1:1	Methyl methacrylate	FeBr_2_	46.0; 20.7	[MMA]_0_/[FeBr_2_]_0_/[EBPA]_0_/[Na_2_CO_3_]_0_ = 200:1:1:2; MMA/DES (*v*/*v*) = 200:1, 60 °C	[80]
Tetrabutylammonium bromide/ Ethylene glycol, 1:2	Methyl methacrylate	FeBr_2_	8.5; 61.1	[MMA]_0_/[FeBr_2_]_0_/[EBPA]_0_/[Na_2_CO_3_]_0_ = 200:1:1:2; MMA/DES (*v*/*v*) = 200:1, 60 °C	[80]
Tetrabutylammonium bromide/ Glycerol, 1:2	Methyl methacrylate	FeBr_2_	5.0; 80.6	[MMA]_0_/[FeBr_2_]_0_/[EBPA]_0_/[Na_2_CO_3_]_0_ = 200:1:1:2; MMA/DES (*v*/*v*) = 200:1, 60 °C	[80]
1,5,7-Triaza-bicyclo[4.4.0]-dec-5-ene/Methanesulfonic acid, 0.1:1.5	ε-caprolactane	Benzyl alcohol (0.15 mmol)	2; 99 (Yield)		[81]
1,5,7-Triaza-bicyclo[4.4.0]dec-5-ene/Methanesulfonic acid, 0.1:1.5	ε-caprolactane	Benzyl alcohol (0.3 mmol)	2; 99		[81]
1,5,7-Triaza-bicyclo[4.4.0]dec-5-ene/Methanesulfonic acid, 0.05:0.75	ε-caprolactane	Benzyl alcohol (0.15 mmol)	2; 99		[81]
1,5,7-Triaza-bicyclo[4.4.0]dec-5-ene/Methanesulfonic acid, 0.05:0.75	ε-caprolactane	Benzyl alcohol (0.3 mmol)	2; 99		[81]
1,5,7-Triaza-bicyclo[4.4.0]dec-5-ene/Methanesulfonic acid, 0.1:1.5	ε-caprolactane		2; 98		[81]
1,5,7-Triaza-bicyclo[4.4.0]dec-5-ene/Methanesulfonic acid, 0.5:1.5	ε-caprolactane		2; 98		[81]
1,8-Octanediol/ Choline chloride, 3:1		Citric acid			[82]
1,8-Octanediol/ Tetraethylammonium bromide, 3:1		Citric acid			[82]
1,8-Octanediol/ Hexadecyltrimethyl-ammonium bromide, 3:1		Citric acid			[82]
1,8-Octanediol/ Methyltriphenyl-phosphonium bromide, 3:1; 3:0.75		Citric acid			[82]
1,8-Octanediol/ Lidocaine, 1:1		Citric acid			[83]
1,8-Octanediol/ Lidocaine, 2:1		Citric acid			[83]
1,8-Octanediol/ Lidocaine, 3:1		Citric acid			[83]
Choline chloride/Urea, 1:2	HEA: 2-hydroxy-ethylacrylate	SARA agent (Na_2_S_2_O_4_)	28-47 min; 16-73	[HEA]0/[HEBiB]0/[_Na2S2O4_]0/[CuBr2]0/[Me_6_TREN]0=DP/0.5/0.1/0.125; [HEA]0/[reline] = 2/1 (*v*/*v*); T = 30 °C (DP (100–1000), CuBr_2_ (1000–100 ppm)	[84]
Choline chloride/Urea, 1:2	HEA: 2-hydroxy-ethylacrylate	SARA agent (Na_2_S_2_O_4_)	2.7; 44	[monomer]0/[HEBiB]0/[Cu (0)]0/[CuBr_2_]0/[ligand]0 = DP/Cu(0) wire/ [CuBr_2_] 0/[ligand]0; Cu(0) wire: l = 5 cm and d = 1 mm; solvent: reline; T = 30 °C; [CuBr_2_]0/ [TPMA]0 = 0.1/0.3; [monomer]0/[reline] = 2/1 (*v*/*v*); DP = 200	[84]
Choline chloride/Urea, 1:2	HEMA: 2-hydroxyethyl methacrylate		7.8; 34	[monomer]0/[HEBiB]0/[Cu (0)]0/[CuBr_2_]0/[ligand]0 = DP/Cu(0) wire/ [CuBr_2_]0/[ligand]0; Cu(0) wire: l = 5 cm and d = 1 mm; solvent: reline; T = 30 °C; [CuBr_2_]0/[TPMA]0 = 0.1/0.3; [monomer]0/[reline] = 2/1 (*v*/*v*); DP = 200	[84]
Choline chloride/Urea, 1:2	AMPTMA: 3-acryl-amidopropyl-triethylammonium chloride		0.7; 83	monomer]0/[HEBiB]0/[Cu (0)]0/[CuBr_2_]0/[ligand]0 = DP/Cu(0) wire/ [CuBr_2_]0/[ligand]0; Cu(0) wire: l = 5 cm and d = 1 mm; solvent: reline; T = 30 °C; [CuBr_2_]0/[Me_6_TREN]0 = 0.5/1.5; [AMPTMA]0/[reline] = 1/1 (*v*/*v*); DP = 100	[84]
Choline chloride/Urea, 1:2	AMPTMA: 3-acrylamidopropyl-triethylammonium chloride (75 wt% aq.)		3.1; 87	monomer]0/[HEBiB]0/[Cu(0)]0/[CuBr_2_]0/[ligand]0 = DP/Cu(0) wire/ [CuBr_2_]0/[ligand]0; Cu(0) wire: l = 5 cm and d = 1 mm; solvent: reline; T = 30 °C; [CuBr_2_]0/[Me_6_TREN]0 = 0.5/1.5; [AMPTMA 75 wt% aq]0 = 1.45 M; DP = 150	[84]
Choline chloride/Urea, 1:2	HEA: 2-hydroxyethyl-acrylate	SARA agent (Na_2_S_2_O_4_)	6.5; 81	FR(Na_2_S_2_O_4_) = 50 nmol/min; [HEA]0/[HEBiB]0 = 200; [HEA]0/[reline]0 = 2/1 (*v*/*v*); [CuBr_2_]_0_/[TPMA]_0_ = 0.1/0.3	[84]
Choline chloride/Urea, 1:2	HEA: 2-hydroxyethyl-acrylate	SARA agent (Na_2_S_2_O_4_)	5.0; 77	FR(Na_2_S_2_O_4_) = 50 nmol/min; [HEA]0/[HEBiB]0 = 200; [HEA]0/[reline]0 = 2/1 (*v*/*v*); [CuBr_2_]_0_/[TPMA]_0_ = 0.1/0.6	[84]
Choline chloride/Urea, 1:2	HEA: 2-hydroxyethyl-acrylate	SARA agent (Na_2_S_2_O_4_)	5.8; 53	FR(Na_2_S_2_O_4_) = 50 nmol/min; [HEA]0/[HEBiB]0 = 200; [HEA]0/[reline]0 = 2/1 (*v*/*v*); [CuBr_2_]_0_/[TPMA]_0_ = 0.3/0.9	[84]
Choline chloride/Urea, 1:2	HEA: 2-hydroxyethyl-acrylate	SARA agent (Na_2_S_2_O_4_)	4.5; 46	FR(Na_2_S_2_O_4_) = 50 nmol/min; [HEA]0/[HEBiB]0 = 200; [HEA]0/[reline]0 = 2/1 (*v*/*v*); [CuBr_2_]_0_/[TPMA]_0_ = 0.3/1.8	[84]
Choline chloride/ Ethylene glycol, 1:2	Methylene blue			Electropolymerization	[85,86]
Betaine/ Mannose, 5:2	Catechin		-; 36–83	Laccase catalysed polymerization	[87]
Choline chloride/Ethylene glycol, 1:2	Catechin		-; 68–92	Laccase catalysed polymerization	[87]
Choline chloride/Glycerol, 1.2	Catechin		-; 72	Laccase catalysed polymerization	[87]
Choline chloride/Urea, 1:2	Acrylamide	catalytic system horseradish peroxidase /H_2_O_2_/2,4-pentanedione	-; >90	Enzyme-mediated free radical polymerization	[88]
Choline chloride/Glycerol, 1:2	Acrylamide	catalytic system horseradish peroxidase /H_2_O_2_/2,4-pentanedione	-; >99	Enzyme-mediated free radical polymerization	[88]
Choline chloride/Fructose, 2:1	2-hydroxyethyl-methacrylate	Indomethacin (in ethanol)		Self-polymerization	[78]
Choline chloride/Orcinol, 1:1.5	2-hydroxyethyl-methacrylate			Self-polymerization	[79]
Choline chloride/Ethylene glycol, 1:2	2-hydroxyethyl methacrylate (HEMA) and poly(ethylene glycol)diacrylate (PEGDA).	2-hydroxy-2-methylpropiophenone photoinitiator		Photopolymerization	[77]
Choline chloride/Ethylene glycol, 1:2	Acrylic acid	Azobisisobutyronitrile and 1-vinylimidazole		Chemical polymerization on the surface of silica particles	[89]
Choline chloride/Ethylene glycol, 1:2	Butyl methacrylate	Cosolvent: 1-butyl-3-methyl-imidazolium tetrafluoro-borate		Synthesis of macroporous poly(HIPEs)–high internal phase emulsions	[90]
Choline chloride/Urea, 1:2	Methylmethacrylate; Stearyl methacrylate; Lauryl acrylate	Different crosslinker: Ethylene glycol dimethyl-acrylate, butanediol diacrylate for acrylates		Synthesis of macroporous poly(HIPEs)	[91]
Choline chloride/Urea, 1:2	Styrene				[92]
Choline chloride/Glycerol, 1:2	Styrene				[92]
